# 

**Published:** 1897-06-05

**Authors:** 


					rv
AtikUliUi'lLLt PUSTT^e^ore tiEliT. .) r\p. 5th, 1897.
OADBURY'S ,^o ^ CADBURY'S
COCOA |p| COCOA
" The standard of highest purity at present Hk 1 NQ ALKALIES USED,
attainable."?Lancet.
ISSTlfS/SSr A 1 - 1 " ' ~-NWMLkM..immcH hosfi.tal
No.^ Yol, XXII. [_a Newspaper J Saturday,
June 5th, 1897.
[Subscnption^ierms,J pKiICE TWOPENCE.

				

